# Celiac Disease and Gallbladder: Pathophysiological Aspects and Clinical Issues

**DOI:** 10.3390/nu14204379

**Published:** 2022-10-19

**Authors:** Dimitri Poddighe, Kuanysh Dossybayeva, Diyora Abdukhakimova, Lyudmila Akhmaltdinova, Aigul Ibrayeva

**Affiliations:** 1 Department of Medicine, Nazarbayev University School of Medicine, Nur-Sultan 010000, Kazakhstan; 2 National Research Center for Maternal and Child Health, Clinical Academic Department of Pediatrics, University Medical Center, Nur-Sultan 010000, Kazakhstan; 3 National Research Cardiac Surgery Center, Nur-Sultan 010000, Kazakhstan; 4 National Research Center for Maternal and Child Health, Program of Pediatric Gastroenterology, Clinical Academic Department of Pediatrics, University Medical Center, Nur-Sultan 010000, Kazakhstan

**Keywords:** gallbladder, celiac disease, gallbladder dysmotility, cholecystokinin, gluten-free diet, cholecystitis, gallstones

## Abstract

**Background**: Celiac Disease (CD) is an immune-mediated disorder which primarily affects the small intestine; however, extra-intestinal organs are often affected by the pathological process, too. As regards the digestive system, liver alterations in CD patients have been widely described, which can also extend to the biliary tract. Notably, gallbladder function can be altered in CD patients. In this review, we specifically analyze and summarize the main pathophysiological aspects and clinical evidence of gallbladder dysfunction in CD patients, in order to discuss the potential medical complications and clinical research gaps. In addition to some perturbations of bile composition, CD patients can develop gallbladder dysmotility, which mainly expresses with an impaired emptying during the digestive phase. The main pathophysiological determinant is a perturbation of cholecystokinin secretion by the specific duodenal enteroendocrine cells in response to the appropriate nutrient stimulation in CD patients. This situation appears to be reversible with a gluten-free diet in most cases. Despite this gallbladder impairment, CD patients do not seem to be more predisposed to gallbladder complications, such as calculous and acalculous cholecystitis. However, very few clinical studies have actively investigated these clinical aspects, which may not be completely evidenced so far; alternatively, the substantial improvements in the last two decades regarding CD diagnosis, which have reduced the diagnostic delay (and related dietary treatment), may have lessened the potential clinical consequences of CD-related gallbladder dysfunction. Specific clinical studies focused on these aspects are needed for a better understanding of the clinical implications of gallbladder alterations in CD patients.

## 1. Introduction

Celiac disease (CD) is an immune-mediated disorder triggered by gluten intake in a minority of individuals harboring a specific genetic HLA-DQ background. Indeed, all patients developing CD basically possess one or more allelic variants related to MHC-DQ2 and/or MHC-DQ8 heterodimers [1]. However, the most relevant alleles for CD predisposition belong to HLA-DQB1, namely HLA-DQB1*02 and, to a lesser extent (in terms of allelic frequency in CD patients), HLA-DQB1*0302 [2,3]. Notably, this genetic background is necessary, but not sufficient, to develop CD upon gluten dietary exposure. Indeed, only 3–4% of these HLA-DQ-predisposed people will be diagnosed with CD over their lives, which means that additional (epi)genetic and/or environmental factors play a substantial role in CD etiopathogenesis at the individual level [4,5].

The primary target of CD is the gut, and indeed, the diagnostic hallmark of CD is the demonstration of gluten-sensitive enteropathy characterized by intraepithelial lymphocyte infiltration and variable degrees of villous atrophy of the small intestine; [1,4] however, CD is a systemic disorder, since many other tissues and organs can be affected in >50% of patients [6].

Among the extra-intestinal targets of CD, the liver is one of the most frequently affected organs. Unexplained hypertransaminasemia with non-specific histologic hepatic changes is the most common hepatic presentation of CD; such a “cryptogenic” liver disorder (ranging from a mild to severe liver dysfunction) often leads to CD diagnosis in silent (asymptomatic) patients and disappears within 1 year of gluten-free diet (GFD) in most cases. Conversely, specific autoimmune liver diseases, such as autoimmune hepatitis and autoimmune cholangitis (which are more frequently diagnosed in CD patients than in the general population), are not gluten sensitive. Recently, CD has been proposed to also be a risk factor for non-alcoholic fatty liver disease [6,7,8].

In addition to the liver, the biliary tract can also be specifically affected in CD patients; in detail, gallbladder function can be altered in these patients. In this minireview, we summarize the main pathophysiological and clinical evidence on this matter and discuss the potential medical consequences in CD patients.

## 2. Overview of Gallbladder Anatomy and Function

The main function of the gallbladder is to accumulate the bile to be poured into the duodenum during the digestive process. However, absorption and secretion processes also occur during the permanence of hepatic bile in the gallbladder; in general, the mucosa can absorb water, sodium, cholesterol, phospholipids, and hydrophilic proteins, whereas it can secrete mucins, hydrogen/chloride ions, and probably immunoglobulins and calcium. The most important effect of these processes is a greater concentration of bile acids in the gallbladder bile compared to hepatic bile [9].

Bile acids are the major lipid components of bile, are synthetized from cholesterol in the liver, and are subsequently conjugated to taurine or glycine, which increases their solubility. Immediately after their synthesis, these (primary) biliary acids are secreted into the bile then concentrated and stored in the gallbladder. Upon food intake, the gallbladder releases the bile into the duodenum, where biliary acids support the digestion and absorption of lipids and fat-soluble vitamins [10].

The gallbladder is located between both hepatic lobes and consists of three anatomic parts: the fundus, corpus, and infundibulum. The gallbladder ends in the cystic duct, which drains into the common bile duct without a sphincteric structure. The common bile duct courses through the head of the pancreas and ends with the sphincter of Oddi, which penetrates the duodenal wall where it forms the ampulla of Vater and opens into the duodenal lumen. The gallbladder wall is formed by a mucosa (with a single layer of epithelial cells and its lamina propria), a single layer of muscle (similar to the muscularis mucosa of the gastrointestinal tract), and a serosal layer [11]. The single muscle layer is innervated by the vagal and splanchnic nerves, which synapse with intramural neurons. Indeed, the biliary tract and, in detail, the gallbladder are functionally integrated with the digestive tract by neuro-hormonal mechanisms in the fasting and digestive phases. In the fasting or inter-digestive phase, the entry of bile occurs through both passive and active mechanisms; the active relaxation allowing the gallbladder to receive the incoming bile is mediated by adrenergic and non-adrenergic fibers. During the three phases of the digestive period (cephalic, antral, and intestinal), the gallbladder is subjected to strong contractions mediated by the aforementioned nerves, but especially by the gut hormone cholecystokinin (CCK). Concomitantly, the sphincter of Oddi becomes relaxed, which allows bile to flow into the duodenum [11,12]. Nervous fibers mainly mediate the cephalic and antral gallbladder emptying, whereas CCK basically induces gallbladder contraction during the intestinal phase, mostly by acting directly on cholinergic neurons. CCK is produced by the I-cells of the intestinal mucosa, which shows the highest number/density in the duodenum and the proximal jejunum. These cells have an apical membrane in direct contact with the intestinal lumen, so they can sense the luminal content. Indeed, the release of CCK is affected by the meal content and, in detail, stimulated by protein- and fat-rich food. The basal cell region, which is close to capillaries, contains the secretory granules, where a mixture of CCK molecular forms with different lengths are stored. Among them, CCK-33 appears to be the predominant form in the human intestine and circulation, where CCK plasma concentrations can reach the picomolar range. Bioactive CCK peptides potently stimulate their target cells by interacting with one of the two CCK receptors expressed on the cell membrane. In detail, the CCK1 receptor is responsible for gallbladder contraction and relaxation of the sphincter Oddi, in addition to other physiological effects, including hepatic bile secretion, pancreatic enzyme secretion and growth, and inhibition of gastric acid secretion and emptying. The CCK2 receptor is primarily expressed in the brain [11,13,14].

## 3. Gallbladder Functioning and Regulation in Celiac Disease

Gallbladder functioning in CD has been intensively investigated starting from about 1970. Indeed, the CD diagnostic hallmark is the damage and flattening of the jejunal/ileal mucosa, which could also impair the endocrine cells located in the mucosal crypts, in addition to the enterocytes. Therefore, such a CD-related intestinal injury could impair nutrients digestion and absorption, both directly (by destroying the enterocytes and, in general, the villous architecture of the intestinal mucosa) and indirectly (by interfering with the production and secretion of gut hormones and peptides implicated in the regulation of intestinal motility and functioning of other organs involved in the digestive process, such as the exocrine pancreas and gallbladder) [15].

Before discussing the impact of CD on gallbladder function, a few studies highlighting some consequences on the bile production and composition in CD patients have to be mentioned [16,17]. Bile flow rate and the secretion of biliary of its main components, such as cholesterol, phospholipids, and bile acids, were reported to be significantly increased in active CD and be normalized after effective GFD. [18] This enhanced secretion of newly synthesized and/or absorbed cholesterol directly into the bile has been associated with a decrease in serum cholesterol concentration in CD patients, [19] which has been observed both in pediatric and adult clinical studies [20,21,22]. However, total plasma cholesterol is often associated with a decrease in HDL-cholesterol and, thus, a higher ratio of total cholesterol to HDL-cholesterol, which can also be ameliorated through GFD [22,23].

Specifically concerning the gallbladder function in CD, in Table 1 we summarize the output of our literature research aimed to extract all the original articles investigating gallbladder motility and/or its main regulator mechanisms, namely CCK production and action, in CD patients. Here, the main study characteristics and findings are also reported. In Table 2, we provide some extracts from the original text of the results and discussion/conclusion included in these articles. Indeed, most of them date back before 2000 and could not be easily retrieved by the readers [16,24,25,26,27,28,29,30,31,32,33,34,35,36,37,38,39,40,41].

Low-Beer et al. first reported absent or impaired gallbladder contraction in CD patients after a fatty meal, despite a normal concentration of the contrast medium administered to perform oral cholecystography. The concomitant study of bile salt turn-over showed a “stagnation of bile in the biliary tree”, supporting the poor contraction of gallbladder after a meal in CD patients [16,17,42]. An experimental study by Di Magno et al. indirectly suggested an impaired secretion of CCK in CD patients after a meal as a main pathophysiological explanation [43]. However, Low-Beer et al. first analyzed the kinetics of plasma CCK after a meal and the concomitant gallbladder response by radioisotope cholecystography, [44] which was described in detail in their seminal paper. They observed an increase of CCK fasting levels, a non-significant rise of CCK levels after a meal and a delayed gallbladder emptying in CD patients compared to controls. Their conclusion was that “at least two factors contribute to the defective gallbladder emptying in patients with coeliac disease. First the rise in serum CCK is less steep than normal. Secondly the gallbladder appears to be less sensitive to the action of CCK, possibly as a result of the abnormally high fasting levels” [24]. This second conclusion was debated by some researchers, [45] but Colombato et al. provided clinical data supporting the main role of deficient CCK production and/or release in CD patients, rather than a resistance of the gallbladder to CCK action. Indeed, these authors observed a normal bile secretory response in CD patients upon exogenous CCK-PZ infusion, which was comparable to that observed in controls [25]. Two additional studies using the pure synthetic CCK analogue caerulein provided conflicting results. Brown et al. reported that their CD patients required a considerably larger dose of caerulein to initiate gallbladder contraction, and its emptying was less complete compared to the controls [31]. Conversely, Masclee et al. found that gallbladder response to cerulein in untreated and treated CD patients was not significantly different from that observed in their controls [33].

Overall, considering all the available and more recent studies included in Table 1 and Table 2, most evidence supports an impaired CCK release into the bloodstream rather than a reduced sensitivity of the gallbladder to CCK. Indeed, several studies also assessed the histological pattern of enteroendocrine cells in the jejunal mucosa and CCK levels of CD patients. Whereas Sjolund et al. and Pietroletti et al. described an increased number of CCK-positive cells in untreated CD patients [26,30], other studies did not confirm this finding and actually found normal or non-significantly reduced CCK-positive enteroendocrine cells in CD patients [27,32,38]. In detail, the study by Calam et al. and especially another one by Deprez et al. further indicated a reduction of CCK plasmatic levels after a meal in CD patients, which can be linked to an impaired production and release of CCK by these specific enteroendocrine cells rather than a decrease in their number [27,37]. Notably, Thimister et al. suggested that such an impaired postprandial CCK release with consequent poor gallbladder contraction in CD patients are not related to general abnormalities in CCK-secreting capacity but to impaired stimulation by nutrients on these enteroendocrine cells [34]. In addition, to further confirm the impairment of CCK secretion in CD patients, Maton et al. provided the first evidence that the impaired CCK release and gallbladder motility can be reversible after GFD [29].

In addition to oral cholecystography and hepatobiliary scintigraphy, “gallbladder inertia” has also been demonstrated by real-time ultrasonography. Delamarre et al. performed it for the first time [28], and all eventual clinical investigations on this matter have relied on this approach since the end of 1990 [34,35,37,40]. Among them, the study by Fraquelli et al. also assessed the somatostatin levels in addition to those of CCK with respect to gallbladder motility. They observed increased circulating levels of somatostatin in untreated CD patients, which they suggested causing increased gallbladder fasting volume, in addition to further supporting an impairment of CCK secretion as a main mechanism leading to the defective gallbladder motor response to a fatty meal [35]. There is no additional research investigating the somatostatin homeostasis and actions in CD specifically; however, several studies confirmed its inhibitory effect on gallbladder contraction in general, even during the postprandial phase. Indeed, it may reduce the plasmatic levels of several gut hormones, including CCK, [46,47,48] which plays a major role in gallbladder contractions and is impaired in CD patients, as discussed above. Regarding the potential role of other gut hormones in CCK plasmatic levels and, thus, the gallbladder dyskinesia in CD, Wahab et al. analyzed peptide YY kinetics in CD patients. They found a strong correlation between plasma peptide YY and plasma CCK, and suggested that the release of CCK in response to digested fat could partially contribute to an increase in peptide YY response to digested fat in these patients [36]. Moreover, Hoentgen et al. concluded that peptide YY can suppress the cephalic phase of postprandial gallbladder emptying but not meal-stimulated maximum emptying, according to their study on both healthy volunteers and untreated CD patients [49].

Most clinical studies included adult CD patients only; however, those few and more recent studies on children seem to confirm the observations made in the adult population overall [30,39,41]. Nousia-Arvanitakis et al. confirmed that plasma CCK release in response to oral nutrients is decreased in untreated CD children; notably, they linked this finding to the presence of intestinal mucosal atrophy in general since a similar response was also observed in patients affected with a different disease, namely cow’s milk protein enteropathy [39]. Das et al. documented gallbladder dysmotility in CD children, which was reversible after GFD [41].

## 4. Is There Any Clinical Implication of Gallbladder Dysfunction in Celiac Disease?

Despite a clear demonstration of CD-related CCK perturbation and gallbladder motor dysfunction, clinical studies focused on gallbladder disease in CD patients are scarce. Notably, most pathophysiological studies were published before 2005, perhaps indicating a decreased interest in this specific matter in the last two decades for some reasons. Probably, as mentioned in the introduction, researchers paid the most attention to the liver complications and/or comorbidity of CD, which can be definitely more relevant in terms of both severity and frequency compared to gallbladder disorders.

Indeed, in addition to the number of studies documenting gallbladder dyskinesia, some research also documented an altered biliary output and bile salt composition in CD patients (as discussed in the previous section), which could theoretically promote the formation of gallstones in these patients. In general, no significant predisposition to gallbladder gallstones and/or calculous cholecystitis has clearly emerged so far [50]. However, very few studies actively investigated these specific aspects in CD, and gallstones-related problems in these patients may have not been completely noticed. Otherwise, we should also consider the great improvement in CD diagnosis and management in the last two decades, which has significantly reduced the diagnostic delay (even in atypical and/or mild forms) and allowed a prompt implementation of GFD, at least in developed countries [51,52,53]; as a consequence, CD-related gallbladder disease may manifest much less frequently than in the case of a longer clinical history without diagnosis and dietary therapy, which can reverse CCK alterations and gallbladder dysmotility, as previously highlighted. This second hypothesis may be partially or indirectly supported by the study by Freeman, who reported 6 cases (20%) of gallstones in a case series including 30 CD patients diagnosed in the elderly. Notably, all of them were reported to have a “severely abnormal flat small intestinal biopsy” [54]. Therefore, one might speculate that these elderly CD patients could have received a more delayed diagnosis compared to younger patients, which may have contributed to a greater detection rate of gallstones.

Indeed, only one case report described a child diagnosed with CD after developing acute calculous cholecystitis [55]. Very recently, Agin et al. investigated the frequency of gallstones in children with CD. Gallstones were detected in 6 out of 120 (5%) of CD children and in 3 out of 100 (3%) controls: no significant difference was reported. However, the timing of abdominal ultrasonography in the study population is not clearly stated, especially with respect to the GFD. Nonetheless, the authors concluded that the early diagnosis and treatment of CD could play a role in preventing the development of gallstones [56].

Gallbladder disease is not only represented by gallstones formation and their potential complications. Indeed, cholecystitis can also occur in the absence of gallstones. Acalculous cholecystitis is more often detected in critically ill patients and less frequently in patients with immune-mediated comorbidities; however, it can even occur in previously healthy individuals. Infections (especially viral) are often detected in the latter category [57], whereas bile stasis, gallbladder ischemia, and inflammatory injury have been variably implicated in (critically) ill patients developing acute acalculous cholecystitis [58]. A recent study also demonstrated a sluggish to paretic gallbladder emptying in response to a small intestinal lipid meal in most critically ill patients developing acute acalculous cholecystitis [59]. Such a gallbladder motor impairment could promote mucosal injury, hypoperfusion, parietal ischemia, and secondary infection of the gallbladder, which are the main mechanisms considered in the pathogenesis of acute acalculous cholecystitis, as mentioned above [60]. However, no well-defined acute acalculous cholecystitis cases have been associated with CD in children and adults, despite the gallbladder dyskinesia in untreated patients. Parfenov et al. described a case of “chronic acalculous cholecystitis” in a 40-year CD patient [61]. This disorder is usually defined as a prolonged inflammatory condition affecting the gallbladder, but the diagnostic criteria are variable and thus not well defined. It is usually considered in patients with a variable combination of symptoms, including recurrent biliary pain ranging from colic to vague discomfort in the upper abdominal right quadrant, and nonspecific complaints, such as nausea, reflux, bloating. Ultrasonography could reveal unspecific findings, including cholelithiasis and gallbladder wall thickening in some cases; notably, clinical studies using gallbladder scintigraphy have pinpointed that a gallbladder affected with chronic cholecystitis has an abnormal contraction, and an impaired/reduced gallbladder ejection fraction is consistent with this diagnosis [62,63,64]. As discussed in the previous section, gallbladder dyskinesia is well demonstrated in CD patients, and the clinical picture of chronic cholecystitis often overlaps with (functional) recurrent abdominal pain and/or dyspeptic syndromes, which are a frequent complaint in CD patients as well [65,66,67]. Therefore, gallbladder dysfunction might contribute to this kind of manifestation in CD patients. However, there are no specific studies in this specific regard that have assessed the frequency of dyspeptic symptoms and/or right upper abdominal pain at the time of CD diagnosis (rather than the prevalence of CD diagnosis in patients with these complaints) and their correlation with gallbladder contractility after a meal. Very recently, Voss et al. analyzed several hepatobiliary disorders in patients affected with chronic intestinal disorders, including CD, by using the ‘UK biobank’ (UKB), which is a population-based cohort study built up in the United Kingdom from 2006 to 2010. Among 2377 individuals with CD, 6.1% received a diagnosis of cholelithiasis, which was significantly higher than controls (3.9%, *p* < 0.001); only 1.1% were diagnosed with unspecified cholecystitis. This value was greater than that observed in controls (0.7%), but the difference did not reach the statistical significance (*p* = 0.071) [68]. Therefore, although one may speculate that gallbladder dysfunction may contribute to some dyspeptic complaints in CD patients, there are no clinical studies investigating this specific aspect, and no final conclusion can be made in this regard.

## 5. Conclusions

In addition to some perturbations of bile composition, CD patients can develop gallbladder dysmotility, characterized by an impaired emptying during the digestive phase. The main pathophysiological determinant is a perturbation of CCK secretion by the specific duodenal enteroendocrine cells in response to the appropriate nutrients stimulation in CD patients. This situation appears to be reversible with GFD in most cases. Despite the gallbladder impairment, CD patients do not seem to be more predisposed to gallbladder complications, such as calculous and acalculous cholecystitis. However, very few clinical studies actively investigated these specific aspects, which may have not been completely evidenced; moreover, the substantial improvements in the last two decades regarding CD diagnosis, which have reduced the diagnostic delay (and related dietary treatment), may have lessened the potential clinical consequences and complications of CD-related gallbladder dysfunction, in terms of additional impairment of digestive processes and/or calculous/acalculous gallbladder disease. Specific clinical studies focused on these aspects are needed for a better understanding of the clinical implications of gallbladder alterations in CD patients. Case–control studies actively investigating gallbladder dysfunction in CD patients could better define the prevalence of this clinical issue in larger cohorts of patients.

## Figures and Tables

**Table 1:** Summary of the main characteristics and findings of clinical studies investigating gallbladder functioning and regulation in CD patients.

Authors, Year [Ref.]	Methods	Stimulus	N of CD Patients [Disease Activity]	CD Patients’ Age (Yrs.)	Control Group (n)	Main Findings
**Low-Beer** et al., **1971** [16]	Oral cholecystography	“fatty meal”	18 [uCD]	Adults (28–78)	Y (36)	-The authors showed that CD patients have a reduced gallbladder contraction/emptying after a meal compared to controls, which can also impair the normal bile salts recirculation.
**Low-Beer** et al., **1975** [24]	Oral chole-cystography + serum CCK concentration by radio- immunoassay	“fatty meal”	10 [uCD]	Adults (M:49)	Y (10)	-This study confirms the impaired motor activity of the gallbladder in untreated CD patients, in response to a meal. -This poor gallbladder response appears to be related to a resistance of this organ to the action of CCK, rather than to an impaired CCK release by the enteroendocrine cells of the small bowel mucosa.
**Colombato** et al., **1977** [25]	Study of “gallbladder evacuation” through analysis of bile secretion and bilirubin output	Mg sulfate solution (i.d) & CCK-PZ (i.v.)	11 [uCD]	Adults (26–62)	Y (10)	-These authors reported a normal bilirubin output following CCK-PZ stimulation in CD patients with atrophy of intestinal mucosa, which was comparable to controls. Therefore, they indirectly suggested an impaired release of CCK (rather than a gallbladder resistance to CCK) in CD patients with damaged intestinal mucosa.
**Sjolund** et al., **1979** [26]	Analysis of hormone-producing cell types in duodenal biopsies by immuno-staining	N/A	18 [uCD?]	Adults (19–60)	Y (24)	-These authors observed that somatostatin cells, GIP cells, and CCK cells were markedly increased in number in the damaged mucosa of CD patients, while the number of secretin cells was slightly reduced. -This enteroendocrine cellular pattern returned to normal, along with the morphology of intestinal mucosa, upon GFD.
**Calam** et al., **1982** [27]	Analysis of amount and type of CCK in duodenal extracts and plasma by radio- immunoassay and gel filtration	N/A	16 [uCD]	Adults (M:2)	Y (13)	-These authors observed a reduced immuno-reactivity of CCK in the intestinal mucosa of CD patients, as well as reduced circulating levels of CCK after a meal, compared to controls. Therefore, they suggested that the impairment of gallbladder function could be related to an impaired production and release of CCK in CD.
**Delamarre** et al., **1984** [28]	Real-time ultrasonography	“fatty meal”	4 [uCD?]	Adults (n/a)	N	-This study confirmed the gallbladder motor dysfunction in CD patients by real-time ultra-sonography, instead of oral cholecystography.
**Maton** et al., **1985** [29]	Study of gallbladder emptying by ^99mTc^-eHIDA HS + serum CCK concentration by radio- immunoassay	“emulsion of arachis oil”	14 [uCD = 6] [nrCD = 2] [gfdCD = 6]	Adults (n/a)	Y (6)	-This study further confirmed that CD patients have an impaired emptying of the gallbladder in response to a fatty meal, by hepatobiliary scintigraphy. -These authors also suggested that such an occurrence can be due to a reduced release of CCK into the circulation, which becomes normalized after GFD.
**Pietroletti** et al., **1986** [30]	Analysis of “endocrine cells” in jejunal biopsies by using a monoclonal antibody to chromogranin	N/A	27 [uCD = 9) [gfdCD = 10] [gcCD = 8]	Children (2–12)	Y (5)	-This study showed an increase of the enteric endocrine cells in children with active CD, in comparison with normal controls. -CD children treated successfully with GFD showed enteroendocrine cell populations similar to the control group.
**Brown** et al., **1987** [31]	Study of gallbladder emptying by ^99mTc^-eHIDA HS	Cerulein-synthetic CCK analogue (i.v)	8 [uCD]	Adults (n/a)	Y (8)	-In this study, CD patients required larger doses of cerulein to initiate gallbladder emptying, and there was a correspondingly longer delay from the start of the infusion until emptying began. A slower gallbladder emptying was also observed. Therefore, these authors suggested that the abnormal gallbladder contraction in CD is not simply because of impaired release of CCK.
**Domschke** et al., **1989** [32]	Analysis of hormone-producing cell types in duodenal biopsies by immuno-staining	N/A	5 [gfdCD]	Adults (n/a)	Y (8)	-No significant differences in mucosal CCK-like immunoreactivity profile was observed between CD patients and controls.
**Masclee** et al., **1991** [33]	Study of gallbladder emptying by ^99mTc^-eHIDA HS + serum CCK concentration by radio- immunoassay	Cerulein-synthetic CCK analogue (i.v)	12 [uCD = 6] [gfdCD = 6]	Adults (38–55)	Y (9)	-These authors reported that gallbladder motor response to cerulein in untreated and treated CD patients was not significantly different from that observed in controls. -Therefore, these authors suggested that the impaired gallbladder contraction in CD patients could result from a reduced endogenous CCK secretion.
**Thimister** et al., **1999** [34]	Real-time ultrasonography + serum CCK concentration by radio- immunoassay	Bombesin (i.v)	13 [uCD = 6] [gfdCD = 7]	Adults (28–55)	Y (7)	-No significant differences in basal and stimulated plasma CCK levels were detected among all the following groups: CD patients with a flat jejunal mucosa, CD patients with an intact intestinal mucosa on GFD, and in controls. -These authors concluded that plasma CCK release and gallbladder contraction in response to bombesin are not reduced in patients with active CD; thus, impaired postprandial CCK release and gallbladder contraction in these patients are not related to abnormal CCK-secreting capacity, but to impaired stimulation by (undigested) nutrients.
**Fraquelli** et al., **1999** [35]	Real-time ultrasonography + serum CCK concentration by radio- immunoassay	“fatty meal”	10 [“at diagnosis and after 18 months of successful gluten-free diet”]	Adults (25–38)	Y (10)	-Gallbladder fasting volume was significantly higher in CD patients at diagnosis than in controls; after a fatty meal, gallbladder emptying was significantly lower in untreated CD patients than in the other two groups. -These authors also observed that an oral fatty meal-induced increase in CCK plasma levels was significantly lower in CD patients at diagnosis than after treatment or in the controls. They suggested a complete restoration of CCK response and normalization of gallbladder contraction after GFD.
**Wahab** et al., **2001** [36]	serum CCK concentration by radio- immunoassay	“pre/un-digested corn oil meal”	22 [uCD = 13] [gfdCD = 9]	Adults (28–55)	Y (15)	-In CD patients, integrated plasma CCK concentrations were significantly increased over basal values in response to predigested fat but not in response to undigested fat.
**Deprez** et al., **2002a** [37]	Real-time ultrasonography + serum CCK concentration by radio- immunoassay	“Liquid polymeric meal”	20 [uCD = 8] [gfdCD = 12: normal mucosa = 6; IELs presence = 8]	Adults (32–55)	Y (9)	-A significant decrease in basal and stimulated CCK plasma was observed in CD patients with a flat mucosa and with the IELs infiltration only, compared to CD patients with healed mucosa upon GFD and volunteers. Thus, they supported a CCK release impairment in CD patients. -Compared to controls, fasting gallbladder volumes were significantly higher in CD patients with mucosal atrophy, who also showed a significantly reduced contraction of gallbladder compared to all groups.
**Deprez** et al., **2002b** [38]	Analysis of CCK-producing cells in duodenal biopsies by immuno-staining and mRNA expression + serum CCK concentration by radio- immunoassay	N/A	19 [uCD = 7] [gfdCD = 12: normal mucosa = 6; IELs presence = 6]	Adults (33–55)	Y (10)	-This study suggested that the defective release of CCK in CD patients is not related to a decrease in the number of CCK cells present in the proximal part of the small intestine but rather to a decrease in CCK synthesis mediated by a decrease in mRNA content.
**Nousia-** **Arvanitakis** et al., **2006** [39]	serum CCK8 concentration by radio- immunoassay	“fatty meal”	24 [uCD]	Children (2–18)	Y (62) * CMPE (12)	-This study supported a poor CCK response to a meal in patients with CD or, in general, patients having flat intestinal mucosa, compared to controls.
**Benini** et al., **2012** [40]	Real-time ultrasonography	meal with 50% lipids	19 [“before (n = 19) and during (n = 14) GFD“]	Adults (M:34)	Y (24)	-Fasting gallbladder volume was significantly larger in CD patients than in healthy controls, as well as residual postprandial gallbladder volume; this gallbladder functional abnormality promptly returned to normal after successful GFD.
**Das** et al., **2021** [41]	^99mTc^-BrIDA HS + Real-time ultrasonography	“fatty meal”	50 [uCD]	Children (M:9)	N	-After strict GFD for a period of 6 months, both ultrasonography and hepatobiliary scintigraphy were concordant in describing the improvement of gallbladder emptying, fasting volume, and postprandial volume in children affected with CD.

* **Abbreviations**: ref., reference number; n, number; yrs., years; Y, yes; N, no; n/a, not available; N/A, not applicable; M, mean; GFD, gluten free diet; uCD, untreated CD patients; nrCD, non-responsive CD patients; gfdCD, CD patients on gluten-free diet; gcCD, CD patients undergoing gluten challenge; CCK, cholecystokinin; PZ, pancreozymin; GIP, gastric inhibitory peptide; ^99mTc^-eHIDA HS, ^99mTc^-labeled diethyl acetanilide-iminodiacetic acid hepatobiliary scintigraphy; ^99mTc^-BrIDAHS, 99mTc-labelled mebrofenin hepatobiliary scintigraphy; CMPE, cow’s milk protein enteropathy; IELs, intraepithelial lymphocytes; i.v., intravenous administration; i.d., intra-duodenal administration.

**Table 2:** Original text extracts from results and discussion/conclusion of the clinical studies investigating gallbladder functioning and regulation in CD patients.

Authors, Year [Ref.]	Results	Conclusions
**Low-Beer** et al., **1971** [16]	-“Area of gallbladder shadow after fat, expressed as percentage of resting area, in coeliac patients and controls” was assessed by the radiologist and related to the degree of gallbladder contraction. -“in coeliac patients the gallbladder area after “fat is significantly greater than normal in relation to the resting value (*p* < 0.01)”.	-“Our finding, in most of the coeliac patients, of a prolonged taurocholate half-life and a decreased recirculation of metabolites of taurocholate is consistent with stagnation of bile in the biliary tree”. -“…in most patients with coeliac disease the gallbladder contracts minimally or not at all in response to a fatty meal”.
**Low-Beer** et al., **1975** [24]	-“Fasting serum CCK levels were significantly raised in patients with CD (mean 1081 pg/mL ± 250 S.E.M.), being higher than in any of the controls (mean 68 pg/mL ± 28 S.E.M.) in nine of the 10 patients (*p* < 0.002). In contrast, the peak serum cholecystokinin levels, and the increments in serum cholecystokinin after fat were both lower in patients with celiac disease, but the difference from control subjects did not reach significant levels”. -“Emptying of the gallbladder was less completed in patients with celiac disease, as judged by the percentage reduction in radioactivity over the gallbladder. After an hour the celiac gallbladder still retained approximately two thirds of its fasting activity, as compared with only one third in controls (*p* < 0.0025)”. -The onset of gallbladder emptying was significantly delayed in celiac patients”.	-“This study confirms the previous findings of a sluggish gallbladder response to food in patients with celiac disease. The poor response, however, does not appear to be due solely to failure of endogenous cholecystokinin release by the abnormal small bowel mucosa, as was previously suggested…” -Peak serum levels after food are normal in patients with celiac disease. The gallbladder itself appears to be resistant to the action of cholecystokinin, and this resistance may in some way to be related to the presence of constantly high levels of this hormone in the blood, the reason for which remains obscure. “
**Colombato** et al., **1977** [25]	-“Following CCK-PZ stimulation, 10 normal control subjects showed a bilirubin increase from 1.92 ± 1.00 to 26.09 ± 7.72 mg/60 min. In 11 patients with intestinal atrophy bilirubin output increased from 0.68 ± 0.45 to 41.09 ± 23.54 mg/60 min. The increase in bilirubin output following CCK-PZ stimulation was not different in the control and patient groups”. -“In 10 normal control subjects the bilirubin output increased from 2.02 ± 0.97 to 35.19 ± 18.45 mg/60 min following instillation of magnesium sulfate, whereas in 11 patients with intestinal atrophy the increase was from 0.76 ± 0.42 to 2.39 ± 1.24 mg/60 min (*p* < 0.001)”.	-“The patients with atrophy of the small-intestinal mucosa in the basal state showed a reduced secretion of bile bilirubin, and in almost half of the cases, bile in the duodenum was totally absent”. -“These results seem to be related to a deficient release or synthesis of endogenous cholecystokinin from a damaged small-intestinal mucosa and seems to confirm that small-intestinal mucosa in celiac sprue is incapable, for whatever reason, of responding to a well-known stimulating mechanism”.
**Sjolund** et al., **1979** [26]	-“Cells displaying somatostatin, GIP or CCK immunoreactivity were fairly numerous in all specimens. The number of these cells was almost doubled in the patients having a flat mucosa”.	-“We found that in the flat mucosa the somatostatin cells, GIP cells, and CCK cells were markedly increased in number, while the number of secretin cells was slightly reduced. The endocrine cell pattern and the intestinal morphology returned to normal upon withdrawal of gluten (four patients)”.
**Calam** et al., **1982** [27]	-“In normal subjects fasting CCK-like immunoreactivity was <0.8 pmol/L, and after a light breakfast increased to 2.0 ± 0.7 (range 1.0 to 4.8) pmol/L; CCK8-like activity accounted for all the increased immunoreactivities. In five of six celiac patients the concentrations of both fasting and postprandial CCK-like immunoreactivity in plasma were undetectable (<0.8 pmol/L)”. -“The study has revealed at least four major forms of immunoreactive CCK in normal human duodenal mucosa. The predominating form (IIN) had the properties of CCK8, […]. All four forms occurred in lower concentration in celiac mucosa compared with normals. In plasma of normal subjects after feeding we were able to demonstrate an increase of CCK8-like immunoreactivity, but significant amounts of CCK-like peptides were not consistently found in the circulation of celiacs after feeding”.	-“We conclude that diminished production and release of CCK could account for the impaired pancreatic and gall bladder responses to intraluminal stimuli in celiac disease”. -“The results raise the possibility that the decreased postprandial circulating CCK in celiacs is a direct consequence of diminished tissue stores of hormone”.
**Delamarre** et al., **1984** [28]	-“In our patients, GB volumes did not significantly decrease after fatty meal. In particular, residual GB volumes 30 min after ingestion were 91.5, 86, 93, and 84%, respectively, of pre-stimulation ones (normal values in 21 volunteers: 52.9 ± 4.5%)”.	-“To our knowledge, our cases are the first in which an ultrasonographic method was applied to study GB function in CD” and supported “gallbladder inertia” in CD patients”.
**Maton** et al., **1985** [29]	-“The half-time of gallbladder emptying were 20.4 ± 2.9 min (mean ± SEM) for normals and 22.1 ± 2.8 min in treated patients with celiac disease (NS). In patients with untreated celiac disease half-times were 154.3 ± 10.3 min (*p* < 0.02 vs. normals and treated patients with celiac disease), and in 2 nonresponsive patients, half-times were 40.7 and 37.3 min”. -“Integrated plasma cholecystokinin responses were 473 ± 87 and 436 ± 137 pm/L/30 min in normals and treated patients with celiac disease (NS). In untreated patients with celiac disease values 322 pmol/L/30 min’. In untreated patients with celiac diseases the values were 16 ± 9 pmoI/L/30 min-’ (*p* < 0.001 vs. normals and treated patients with celiac disease), and in nonresponsive patients values were 442 and 322 pmol/L/30 min”.	-“These data demonstrate that, in untreated celiac disease, there is a failure of the gallbladder to empty in response to a fatty meal, and this is associated with a failure to release CCKs into the circulation. After adequate treatment with a gluten free diet, the meal liberates CCK and causes gallbladder contraction. Our results also suggest that, in nonresponsive celiac disease, there is slightly impaired gallbladder emptying associated with a mild reduction in circulating CCK…” -“We conclude that there is a reversible defect of gallbladder emptying and cholecystokinin release in celiac disease”.
**Pietroletti** et al., **1986** [30]	-“The density of endocrine cells for each group is shown in the Table. The analysis of variance (f-test) revealed statistically significant differences in the results for each group (*p* < 0.01). A multiple comparison was therefore carried out using Scheffe’s test. All four quantitative methods used showed that the active coeliacs (group a) and the coeliacs receiving gluten-challenge (group c) had significantly more endocrine cells than the normal controls. -“Comparison between the morphological features of the disease, villous atrophy and lymphocytic infiltration, and endocrine cell density revealed no obvious relationship”.	-“In the present study, all the methods of quantification used showed a significant increase in the enteric endocrine cells of coeliacs with active disease, in comparison with normal controls. Coeliac children who had been treated successfully with a gluten-free diet, however, showed a gut endocrine cell population similar to that seen in the control group”. -“In conclusion, it seems that a complex modification of endocrine cell turnover takes place in active coeliac disease. These changes, which disappear after effective therapy, may give rise to the reported functional disturbances in gut hormone release”.
**Brown** et al., **1987** [31]	-“Patients with coeliac disease required a considerably larger dose of caerulein to initiate gall bladder emptying (3.80 ± 1.08 v 1.49 ± 0.56 ng/kg, *p* < 0.02), and there was a correspondingly longer delay from the start of the infusion until emptying began. The speed of gall bladder emptying tended to be slower than normal in patients with coeliac disease, but this was not statistically significant”. -“By the end of the 60-min infusion of caerulein, gall bladder emptying was less complete in the patients than in the controls (percentage emptying at end of caerulein infusion 34.6 ± 9.9 v 61.5 ± 7.5, *p* < 0.02)”.	-“These results suggest that the abnormal gall bladder contraction in coeliac disease is not simply because of impaired release of cholecystokinin. Although mechanical factors secondary to the increased gall bladder size in patients with coeliac disease might to some extent account for the findings, the alternative explanation is that the gall bladder muscle is for some reason resistant to the action of cholecystokinetic agents”.
**Domschke** et al., **1989** [32]	-“The mucosal CCK-like immunoreactivity showed no peculiar profile in coeliac sprue. In general, the duodenal CCK-concentrations of coeliac sprue patients tended to be lower than control values. The level of significance (*p* < 0.05) was, however, not reached”.	-“Although the CCK measurements were made at the site of maximal involvement with coeliac disease and the morphology of duodenum and proximal jejunal segments had not reverted toward normal, mucosal CCK concentrations of our coeliac sprue patients were not significantly different from controls. They showed, however, a tendency to lowered mucosal levels”.
**Masclee** et al., **1991** [33]	-“Both in the patients and in the controls infusion of stepwise increasing doses of cerulein, in the range of 1–16 ng/kg/h, induced dose-related changes in plasma CCK-like immunoreactivity (CCK-LI) (r = 0.99; *p* < 0.001) and gallbladder emptying (r > 0.97; *p* < 0.01). Plasma CCK-LI and gallbladder responses were not significantly different among untreated coeliac patients, treated coeliac patients, and controls”.	-“Gallbladder sensitivity to cerulein in untreated and treated coeliac patients was not significantly different from that in controls. It is concluded that the abnormally decreased gallbladder contraction in coeliac patients is the result of a reduced endogenous CCK secretion and not of a lack of end-organ responsiveness to CCK”.
**Thimister** et al., **1999** [34]	-“Basal plasma CCK levels in celiac patients with a flat jejunal mucosa (2.8 ± 0.2 pM), in patients with an intact intestinal mucosa while on a glute n-free die t (3.3 ± 0.2 pM) and in controls (2.8 ± 0.2 pM) were not significantly different from each other”. -“Plasma CCK increments in response to these stepwise increasing doses of BBS were not significantly different among patients and normal subjects” -“The average slopes of the linear regression of plasma CCK increments and log BBS doses […] were not significantly different among the three groups, indicating similar dose-response relationships between healthy subjects and patients with celiac disease”. -“Fasting gallbladder volume was 31.1 ± 5.5 mL in untreated patients, 44.2 ± 7.9 mL in treated patients, and 26.5 ± 2.8 mL in healthy volunteers (NS). -“The degree of gallbladder contraction at the end of each BBS dose was not significantly different among celiac patients and healthy controls, [as well as] the average slopes of the linear regression of decrease s in gallbladder volume and log BBS doses…indicating similar dose ± response relationships”.	-“The present study shows that intravenous infusion of BBS markedly and dose dependently stimulates plasma CCK release and gallbladder contraction in celiac patients with a flat jejunal mucosa. Plasma CCK and gallbladder responses to bombesin in the patients with a flat jejunal mucosa were comparable to the response s found in healthy subjects and in celiac patients with a normal intestinal mucosa while on a gluten free diet”. -“Because CCK is the principal mediator of bombesin-stimulated gallbladder contraction, this suggests that gallbladder sensitivity to circulating endogenous CCK is not impaired”. -“In conclusion, plasma CCK release and gallbladder contraction in response to increasing dose s of intravenously administered BBS are not reduced in patients with celiac disease and a flat jejunal mucosa when compared to healthy subjects. Thus, the impaired postprandial CCK release and gallbladder contraction in these patients are not related to abnormalities in CCK-secreting capacity but to impaired stimulation by (undigested) nutrients”.
**Fraquelli** et al., **1999** [35]	-“GB fasting volume was significantly higher in celiac patients at diagnosis than in controls (*p* = 0.021) and at a borderline level of significance after GFD (*p* = 0.059). After the fatty meal, GB EF% was significantly lower in the newly diagnosed patients than in the other two groups (*p* = 0.005 and *p* = 0.007, respectively) and GB residual volume was significantly higher (*p* = 0.005 and *p* = 0.01, respectively)”. -“Celiac patients at diagnosis had a significantly greater mean overall emptying volume than controls (16.97 vs. 7.20 mL, respectively; *p* = 0.0007). […] Successful GFD significantly reduced the overall emptying volume (16.97 vs. 9.16 mL, *p* = 0.0057)”. -“… basal CCK plasma levels were comparable in celiac patients before and after GFD and in controls. After a fatty meal, patients at diagnosis had significantly lower CCK peak values and areas under the concentration time curves (CCK AUC) than patients after GFD and controls (*p* = 0.009 and *p* = 0.028, and *p* = 0.028 for both comparisons, respectively)”. -“In all subjects GB EF% correlated with CCK peak values (r_s_ = 0.68, *p* = 0.002) and CCK AUC (r_s_ = 0.81, *p* = 0.001)”.	-“The oral fatty meal-induced increase in CCK plasma levels, as shown by CCK peak and AUC, was significantly lower in our celiac patients at diagnosis than after treatment or in the controls. […] the low CCK peak value and small CCK integrated secretion may explain the lower GB EF% observed in the celiac patients before treatment compared with the other two groups. The complete restoration of CCK response to a fatty meal and normalization of GB contraction after successful long-term GFD…”. -“Overall, in untreated celiac patients increased SS circulating levels seem to cause increased GB fasting volume, whereas derangement of CCK secretion by atrophic small intestinal mucosa could be the main mechanism underlying the significantly defective GB motor response to a fatty meal”.
**Wahab** et al., **2001** [36]	-“Basal PYY was increased in untreated celiac patients (N = 13) compared to patients on a gluten free diet (N = 9) [15.6 (11.8–27.0) pM vs. 12.2 (10.1–13.1) pM; *p* < 0.05] and compared to control subjects (N = 15) [9.5 (8.3–10.4) pM; *p* < 0.001]. Integrated PYY in response to intraduodenally infused predigested fat (1071 ± 293 pM 80 min) was significantly (*p* < 0.05) greater than in response to undigested fat (322 ± 223 pM 80 min) in six untreated celiacs”. - “Integrated plasma CCK concentrations were significantly increased over basal values in response to predigested fat (113 ± 32 pM; *p* = 0.02), but not in response to undigested fat (51 ± 41 pM; NS). Plasma CCK concentrations were significantly correlated with plasma PYY concentrations (r = 0.79; *p* = 0.001)”.	-“The strong correlation between plasma PYY and plasma CCK demonstrated in the present study supports this hypothesis and it suggests that the release of CCK in response to digested fat contributes at least in part to the increased PYY response to digested fat in celiac patients”.
**Deprez** et al., **2002a** [37]	-“A significant decrease in basal CCK plasma was observed in group C celiac patients with a flat mucosa (0.6 [0.3–1.3] pmol/L) and in group B patients with the infiltrative type (0.4 [0.2–0.7] pmol/L), compared with the patients with a totally normalised mucosa (1.6 [1.0–2.4] pmol/L) and with the volunteers (1.0 [0.7–1.4] pmol/L) (*p* < 0.005)”. -“Although no differences could be seen between the two test meals, a significantly decreased CCK release was observed in groups B and C patients (*p* < 0.05). […] Peak values of CCK immunoreactivity were significantly decreased in celiac patients with a flat mucosa (group C) and the infiltrative type of mucosa (group B) (*p* < 0.0001)” -“Initial fasting gallbladder volumes were 19 ± 2, 23 ± 3 and 37 ± 6 cm^3^ in control subjects, groups B and C patients, respectively. Group C fasting gallbladder volumes were significantly higher compared with the control subjects (*p* < 0.05). Significant contraction of gallbladder was observed in all groups except in group C patients with a destructive mucosa. Postprandial values were 8 ± 1 cm3 in control subjects (*p* < 0.001, compared with fasting volumes), 12 ± 1 cm^3^ in group B (*p* < 0.005) and 24 ± 4 cm^3^(NS) in group C”.	-“Our study confirmed that CCK release is impaired in untreated celiac patients. Although previous reports demonstrated a recovery of normal CCK release in patients under a gluten-free diet, we were able to show that this recovery needs full restoration of the celiac duodenal mucosa. A defective release was indeed still present in patients whose mucosa displayed a significant IEL infiltrate. Only patients without any atrophy or subatrophy and without any increased IEL infiltrate had a basal and postprandial CCK release similar to the control group. These results favour the hypothesis of an interaction between IELs and CCK cells”. -“In conclusion, we demonstrated that the intraepithelial lymphocytic infiltrate observed in the intestinal mucosa of patients with celiac disease is associated with decreased plasma CCK levels. CCK postprandial plasma secretion was not improved by the administration of a predigested standard meal. Impaired CCK release in celiac disease is therefore not due to reduced protein hydrolysis in the duodenal lumen, that was previously linked to mucosal atrophy. Some inhibitory mechanism could be involved in the CCK cell dysfunction observed in celiac patients presenting with lesser degrees of disease activity”.
**Deprez** et al., **2002b** [38]	-“CCK cell size did not change, and CCK cells were found in crypts and villi both in controls and patients with an infiltrative pattern or a normalized mucosa, and throughout the crypts of patients with atrophic mucosa. A nonsignificant decrease in the number of CCK cells, in the epithelial surface and in the ratio of CCK cells to epithelial surface was observed in group A (patients with atrophic mucosa)”. -“Between-group comparison showed a significant decrease in CCK concentration in patients from group A with atrophic changes (*p* = 0.006), but surprisingly also in patients from group B with an infiltrative pattern (*p* = 0.01)”. -“Significantly lower concentrations of CCK mRNA were seen in patients with a destructive-type mucosa compared with controls and treated patients with a normal mucosa (CCK}RPL19 ratio, *p* = 0.03). Interestingly, a similar decrease was also seen in the duodenal biopsies with an infiltrative pattern (*p* = 0.05)”.	-“The present studies provide evidence that the defective release of CCK, observed in patients with coeliac disease, is not related to a decrease in the number of CCK cells present in the proximal part of the small intestine, but rather to a decrease in CCK synthesis mediated by a decrease in mRNA content”. -“In conclusion, our results confirm an impaired upper intestinal endocrine function in coeliac disease. We demonstrated a decrease in duodenal CCK concentrations and a reduction in CCK mRNA transcripts without significant changes in the number of CCK expressing cells, especially in patients with high IEL counts. Reduced expression of the CCK gene could, therefore, be related to suppressive factors induced by the inflammatory infiltrate”.
**Nousia-Arvanitakis** et al., **2006** [39]	-“Plasma octapeptide CCK8 values obtained from 12 children with CMPE and 24 patients with CD at a time when they were on milk-free and gluten-free diet, respectively, and had normal intestinal mucosa, […]. Mean fasting values (T ± SD) were 0.7725 ± 0.3209 and 1.0092 ± 0.6304 pmol/L in CMPE and CD, respectively. The values were comparable in the 2 groups and the controls (0.7317 ± 0.6909) pmol/L (*p* > 0.05). Mean postprandial values were 1.8492 ± 0.6173 and 2.5792 ± 1.6243 pmol/L in CMPE and CD, respectively. Again, there was no statistical difference between the 2 groups and the controls (2.0084 ± 1.8570 pmol/L; *p* > 0.05). There was significant difference, however, between fasting and postprandial values in both groups and the controls (*p* < 0.001)”. -“Plasma CCK values obtained from patients with villous atrophy…Mean fasting values were 0.6475 ± 0.3264 and 0.7383 ± 0.5186 pmol/L in CMPE and CD, respectively. They were not statistically different from those of the control group (0.7317 ± 0.6709 pmol/L; *p* > 0.05). Mean postprandial values were 0.7133 ± 0.3019 and 0.8246 ± 0.5132 pmol/L in CMPE and CD, respectively. They were comparable (*p* > 0.05) but statistically different from those of the control group (2.0084 ± 1.8570 pmol/L; *p* = 0.001). There was no significant difference between fasting and postprandial values in CMPE, whereas the difference in CD barely reached statistical significance (*p* = 0.046)”.	-The study has also demonstrated poor CCK response to a meal in patients with CD or CMPE patients having flat intestinal mucosa. The meal-induced increase in CCK plasma levels were significantly lower in patients with flat intestinal mucosa as compared with those having normal mucosa or controls”. -“In conclusion, we have demonstrated that plasma CCK release in response to oral nutrients is decreased in patients having intestinal mucosa atrophy. This may lead to lack of exocrine pancreas stimulation. This study supports the hypothesis that exocrine pancreatic impairment in patients, who have intestinal mucosal atrophy and are not markedly undernourished, may be attributed to reduced CCK secretion from the CCK secreting cells located in the damaged intestinal mucosa rather than pancreatic acinar dysfunction”.
**Benini** et al., **2012** [40]	-“Mean fasting GB volume was significantly larger in CD patients than in healthy controls (32.2 ± 19.1 mL vs. 17.6 ± 5.1 mL, respectively, *p* = 0.0041), as was residual postprandial GB volume (8.5 ± 8.8 mL vs. 2.2 ± 1.7 mL), respectively, *p* = 0.0038)”. -“Fourteen of the 19 CD patients enrolled in the study have been studied twice, before and during GFD. […]GB volume decreased in each individual patient and mean value decreased from 32.1 ± 1.4 mL at baseline to 20.6 ± 9.6 mL (*p* = 0.0549) during GFD, a value similar to that observed in healthy controls (17.6 ± 5.1 mL). Postprandial residual volume also decreased in each individual patient and mean value volume decreased from 8.3 ± 8.9 mL at baseline to 2.4 ± 1.7 mL during GFD (*p* = 0.0134), a value similar to that observed in healthy controls (2.2 ± 1.7 mL)”.	-“Our study confirms a functional alteration of GB motility in CD patients, and a prompt return to normality during GFD”.
**Das** et al., **2021** [41]	-“After a strict GFD for a period of 6 months, repeat HBS and USG were performed to evaluate GBEF in the 8 children who had impaired GBEF by HBS at baseline. Apart from a significant improvement in GBEF on HBS (19% to 74%, *p* < 0.001), the GBEF also improved significantly as assessed by USG parameters after GFD (*p* < 0.001). The fasting GB volume reduced, with significant improvement in postprandial percentage GB volume change compared to baseline pre-GFD values”. -“A subgroup analysis was done comparing children with normal and reduced GBEF (Table 3). Mean delay in diagnosis (6.5 ± 2.0 vs. 2.3 ± 1.2 years, *p* < 0.001) and OCTT (96.2 ± 14 vs. 56.1 ± 12 min, *p* < 0.001) were significantly higher in children with reduced GBEF. […] The delay in diagnosis had a significant negative correlation with baseline GBEF on HBS (r = −0.5, *p* < 0.001) and percentage postprandial GB volume change on USG (r = −0.3, *p* < 0.01), […]”.	-“After ensuring GFD for 6 months, the subgroup of children with reduced GBEF had significant improvement in GBEF parameters as assessed by both HBS (increase to 74% from 19%) and USG (increase to 52% from 24%), indicating that a GFD can reverse the abnormalities of GB motility in children with CD. GFD results in the reconstitution of normal villous architecture with return of normal CCK cell function, leading to an improvement in GB motility”. -“To conclude, GB dysmotility can occur in children with CD, especially if the diagnosis is delayed, but it is reversible with adherence to GFD. GB hypomotility may translate into gallstone disease, with its related complications, in adulthood if CD is left untreated”.

**Abbreviations**: ref., reference number; CCK, cholecystokinin; PZ, pancreozymin; GIP, gastric inhibitory peptide; GB, gallbladder; S.E.M., standard error of the mean; GBEF, gallbladder ejection fraction; CMPE, cow’s milk protein enteropathy; IELs, intraepithelial lymphocytes; i.v., intravenous administration; i.d., intra-duodenal administration; n or N, number.

## Data Availability

Not applicable.

## References

[1] Lindfors K., Ciacci C., Kurppa K., Lundin K.E.A., Makharia G.K., Mearin M.L., Murray J.A., Verdu E.F., Kaukinen K. (2019). Coeliac Disease. Nat. Rev. Dis. Prim..

[2] Espino L., Núñez C. (2021). The HLA Complex and Coeliac Disease. Int. Rev. Cell. Mol. Biol..

[3] Poddighe D., Rebuffi C., De Silvestri A., Capittini C. (2020). Carrier Frequency of HLA-DQB1*02 Allele in Patients Affected with Celiac Disease: A Systematic Review Assessing the Potential Rationale of a Targeted Allelic Genotyping as a First-Line Screening. World J. Gastroenterol..

[4] Lebwohl B., Sanders D.S., Green P.H.R. (2018). Coeliac Disease. Lancet.

[5] Poddighe D., Capittini C. (2021). The Role of HLA in the Association between IgA Deficiency and Celiac Disease. Dis. Markers.

[6] Nardecchia S., Auricchio R., Discepolo V., Troncone R. (2019). Extra-Intestinal Manifestations of Coeliac Disease in Children: Clinical Features and Mechanisms. Front. Pediatr..

[7] Volta U. (2009). Pathogenesis and Clinical Significance of Liver Injury in Celiac Disease. Clin. Rev. Allergy Immunol..

[8] Marciano F., Savoia M., Vajro P. (2016). Celiac Disease-Related Hepatic Injury: Insights into Associated Conditions and Underlying Pathomechanisms. Dig. Liver Dis..

[9] Turumin J.L., Shanturov V.A., Turumina H.E. (2013). The Role of the Gallbladder in Humans. Rev. Gastroenterol. Mex..

[10] di Ciaula A., Garruti G., Baccetto R.L., Molina-Molina E., Bonfrate L., Wang D.Q.-H., Portincasa P. (2017). Bile Acid Physiology. Ann. Hepatol..

[11] Behar J. (2013). Physiology and Pathophysiology of the Biliary Tract: The Gallbladder and Sphincter of Oddi—A Review. ISRN Physiol..

[12] Mawe G.M. (1998). Nerves and Hormones Interact to Control Gallbladder Function. News Physiol. Sci..

[13] Rehfeld J.F. (2021). Cholecystokinin and the Hormone Concept. Endocr. Connect..

[14] Rehfeld J.F. (2004). Clinical Endocrinology and Metabolism. Cholecystokinin. Best. Pract. Res. Clin. Endocrinol. Metab..

[15] Usai-Satta P., Oppia F., Lai M., Cabras F. (2018). Motility Disorders in Celiac Disease and Non-Celiac Gluten Sensitivity: The Impact of a Gluten-Free Diet. Nutrients.

[16] Low-Beer T.S., Heaton K.W., Heaton S.T., Read A.E. (1971). Gallbladder Inertia and Sluggish Enterohepatic Circulation of Bile-Salts in Coeliac Disease. Lancet.

[17] Low-Beer T.S., Heaton K.W., Pomare E.W., Read A.E. (1973). The Effect of Coeliac Disease upon Bile Salts. Gut.

[18] Vuoristo M., Miettinen T.A. (1985). Increased Biliary Lipid Secretion in Celiac Disease. Gastroenterology.

[19] Vuoristo M., Miettinen T.A. (1986). Serum Cholesterol Precursor Sterols in Coeliac Disease: Effects of Gluten Free Diet and Cholestyramine. Gut.

[20] Ciacci C., Cirillo M., Giorgetti G., Alfinito F., Franchi A., di Pietralata M.M., Mazzacca G. (1999). Low Plasma Cholesterol: A Correlate of Nondiagnosed Celiac Disease in Adults with Hypochromic Anemia. Am. J. Gastroenterol..

[21] Jamnik J., Jenkins D.J., El-Sohemy A. (2018). Biomarkers of Cardiometabolic Health and Nutritional Status in Individuals with Positive Celiac Disease Serology. Nutr. Health.

[22] Ciampolini M., Bini S. (1991). Serum Lipids in Celiac Children. J. Pediatr. Gastroenterol. Nutr..

[23] Brar P., Kwon G.Y., Holleran S., Bai D., Tall A.R., Ramakrishnan R., Green P.H.R. (2006). Change in Lipid Profile in Celiac Disease: Beneficial Effect of Gluten-Free Diet. Am. J. Med..

[24] Low-Beer T.S., Harvey R.F., Davies E.R., Read A.F. (1975). Abnormalities of Serum Cholecystokinin and Gallbladder Emptying in Celiac Disease. N. Engl. J. Med..

[25] Colombato L.O., Parodi H., Cantor D. (1977). Biliary Function Studies in Patients with Celiac Sprue. Am. J. Dig. Dis..

[26] Sjölund K., Alumets J., Berg N.O., Håkanson R., Sundler F. (1979). Duodenal Endocrine Cells in Adult Coeliac Disease. Gut.

[27] Calam J., Ellis A., Dockray G.J. (1982). Identification and Measurement of Molecular Variants of Cholecystokinin in Duodenal Mucosa and Plasma. Diminished Concentrations in Patients with Celiac Disease. J. Clin. Investig..

[28] Delamarre J., Capron J.P., Joly J.P., Audebert M., Murat J.L., Remond A., Revert R., Trinez G. (1984). Gallbladder Inertia in Celiac Disease: Ultrasonographic Demonstration. Dig. Dis. Sci..

[29] Maton P.N., Selden A.C., Fitzpatrick M.L., Chadwick V.S. (1985). Defective Gallbladder Emptying and Cholecystokinin Release in Celiac Disease. Reversal by Gluten-Free Diet. Gastroenterology.

[30] Pietroletti R., Bishop A.E., Carlei F., Bonamico M., Lloyd R.V., Wilson B.S., Ceccamea A., Lezoche E., Speranza V., Polak J.M. (1986). Gut Endocrine Cell Population in Coeliac Disease Estimated by Immunocytochemistry Using a Monoclonal Antibody to Chromogranin. Gut.

[31] Brown A.M., Bradshaw M.J., Richardson R., Wheeler J.G., Harvey R.F. (1987). Pathogenesis of the Impaired Gall Bladder Contraction of Coeliac Disease. Gut.

[32] Domschke S., Bloom S.R., Adrian T.E., Lux G., Bryant M.G., Domschke W. (1989). Coeliac Sprue: Abnormalities of the Hormone Profile of Gastroduodenal Mucosa. Scand. J. Gastroenterol..

[33] Masclee A.A., Jansen J.B., Driessen W.M., Geuskens L.M., Lamers C.B. (1991). Gallbladder Sensitivity to Cholecystokinin in Coeliac Disease. Correlation of Gallbladder Contraction with Plasma Cholecystokinin-like Immunoreactivity during Infusion of Cerulein. Scand. J. Gastroenterol..

[34] Thimister P.W., Hopman W.P., Rosenbusch G., Jansen J.B. (1998). Plasma Cholecystokinin and Gallbladder Responses to Increasing Doses of Bombesin in Celiac Disease. Dig. Dis. Sci..

[35] Fraquelli M., Bardella M.T., Peracchi M., Cesana B.M., Bianchi P.A., Conte D. (1999). Gallbladder Emptying and Somatostatin and Cholecystokinin Plasma Levels in Celiac Disease. Am. J. Gastroenterol..

[36] Wahab P.J., Hopman W.P., Jansen J.B. (2001). Basal and Fat-Stimulated Plasma Peptide YY Levels in Celiac Disease. Dig. Dis. Sci..

[37] Deprez P.H., Sempoux C., van Beers B.E., Jouret A., Robert A., Rahier J., Geubel A., Pauwels S., Mainguet P. (2002). Persistent Decreased Plasma Cholecystokinin Levels in Celiac Patients under Gluten-Free Diet: Respective Roles of Histological Changes and Nutrient Hydrolysis. Regul. Pept..

[38] Deprez P.H., Sempoux C., de Saeger C., Rahier J., Mainguet P., Pauwels S., Geubel A. (2002). Expression of Cholecystokinin in the Duodenum of Patients with Coeliac Disease: Respective Role of Atrophy and Lymphocytic Infiltration. Clin. Sci..

[39] Nousia-Arvanitakis S., Fotoulaki M., Tendzidou K., Vassilaki C., Agguridaki C., Karamouzis M. (2006). Subclinical Exocrine Pancreatic Dysfunction Resulting from Decreased Cholecystokinin Secretion in the Presence of Intestinal Villous Atrophy. J. Pediatr. Gastroenterol. Nutr..

[40] Benini F., Mora A., Turini D., Bertolazzi S., Lanzarotto F., Ricci C., Villanacci V., Barbara G., Stanghellini V., Lanzini A. (2012). Slow Gallbladder Emptying Reverts to Normal but Small Intestinal Transit of a Physiological Meal Remains Slow in Celiac Patients during Gluten-Free Diet. Neurogastroenterol. Motil..

[41] Das S., Lal S.B., Venkatesh V., Bhattacharya A., Saxena A., Thapa B.R., Rana S.V. (2021). Gallbladder Motility in Children with Celiac Disease before and after Gluten-Free Diet. Ann. Gastroenterol..

[42] Low-Beer T.S., Heaton K.W., Read A.E. (1970). Gallbladder Inertia in Adult Coeliac Disease. Gut.

[43] DiMagno E.P., Go V.L., Summerskill W.H. (1972). Impaired cholecystokinin-pancreozymin secretion, intraluminal dilution, and maldigestion of fat in sprue. Gastroenterology.

[44] Low-Beer T.S., Harvey R.F., Nolan D., Davies E.R., Read A.E. (1974). Proceedings: Abnormalities of Cholecystokinin Secretion and Gallbladder Emptying in Coeliac Disease. Gut.

[45] DiMagno E.P., Go V.L., Summerskill W.H. (1975). Letter: Gallbladder Function in Nontropical Sprue. N. Engl. J. Med..

[46] Vu M.K., van Oostayen J.A., Biemond I., Masclee A.A. (2001). Effect of Somatostatin on Postprandial Gallbladder Relaxation. Clin. Physiol..

[47] Fisher R.S., Rock E., Levin G., Malmud L. (1987). Effects of Somatostatin on Gallbladder Emptying. Gastroenterology.

[48] Fraquelli M., Pagliarulo M., Colucci A., Paggi S., Conte D. (2003). Gallbladder Motility in Obesity, Diabetes Mellitus and Coeliac Disease. Dig. Liver Dis..

[49] Hoentjen F., Hopman W.P., Jansen J.B. (2001). Effect of Circulating Peptide YY on Gallbladder Emptying in Humans. Scand. J. Gastroenterol..

[50] Wang H.H., Liu M., Li X., Portincasa P., Wang D.Q.-H. (2017). Impaired Intestinal Cholecystokinin Secretion, a Fascinating but Overlooked Link between Coeliac Disease and Cholesterol Gallstone Disease. Eur. J. Clin. Investig..

[51] Makharia G.K., Chauhan A., Singh P., Ahuja V. (2022). Review Article: Epidemiology of Coeliac Disease. Aliment. Pharmacol. Ther..

[52] Villanueva M., Oyarzún A., Leyton B., González M., Navarro E., Canales P., Ossa C., Muñoz M.P., Bascuñán K.A., Araya M. (2020). Changes in Age at Diagnosis and Nutritional Course of Celiac Disease in the Last Two Decades. Nutrients.

[53] Poddighe D., Abdukhakimova D. (2021). Celiac Disease in Asia beyond the Middle East and Indian Subcontinent: Epidemiological Burden and Diagnostic Barriers. World J. Gastroenterol..

[54] Freeman H.J. (1995). Clinical Spectrum of Biopsy-Defined Celiac Disease in the Elderly. Can. J. Gastroenterol..

[55] Bertrand L.G., Ortiz L.O., Chazarra C.T., Blanquer F.A., Septien I.O., Cantó V.E., Cerdá J.J. (2006). Colecistitis aguda litiásica como presentación excepcional de enfermedad celíaca. Anal. Pediatr..

[56] Agin M., Kayar Y. (2021). Gallstone Frequency in Children with Celiac Disease. Cureus.

[57] Poddighe D., Cagnoli G., Mastricci N., Bruni P. (2014). Acute Acalculous Cholecystitis Associated with Severe EBV Hepatitis in an Immunocompetent Child. BMJ Case Rep..

[58] Poddighe D., Sazonov V. (2018). Acute Acalculous Cholecystitis in Children. World J. Gastroenterol..

[59] Plummer M.P., Kar P., Cousins C.E., Hausken T., Lange K., Chapman M.J., Jones K.L., Horowitz M., Deane A.M. (2016). Critical Illness Is Associated With Impaired Gallbladder Emptying as Assessed by 3D Ultrasound. Crit. Care Med..

[60] Barie P.S., Eachempati S.R. (2010). Acute Acalculous Cholecystitis. Gastroenterol. Clin. N. Am..

[61] Parfenov A.I., Dolgasheva G.M., Krums L.M., Bystrovskaia E.V., Sabel’nikova E.A., Gudkova R.B., Vorob’eva N.N., Lishchinskaia A.A. (2011). Asymptomatic celiac disease in patient with chronic acalculous cholecystitis. Eksp. Klin. Gastroenterol..

[62] Lam R., Zakko A., Petrov J.C., Kumar P., Duffy A.J., Muniraj T. (2021). Gallbladder Disorders: A Comprehensive Review. Dis. Mon..

[63] Ziessman H.A. (2001). Cholecystokinin Cholescintigraphy: Clinical Indications and Proper Methodology. Radiol. Clin. N. Am..

[64] Kwatra N.S., Nurko S., Stamoulis C., Falone A.E., Grant F.D., Treves S.T. (2019). Chronic Acalculous Cholecystitis in Children With Biliary Symptoms: Usefulness of Hepatocholescintigraphy. J. Pediatr. Gastroenterol. Nutr..

[65] Aziz I., Simrén M. (2021). The Overlap between Irritable Bowel Syndrome and Organic Gastrointestinal Diseases. Lancet Gastroenterol. Hepatol..

[66] Petrarca L., Nenna R., Mastrogiorgio G., Florio M., Brighi M., Pontone S. (2014). Dyspepsia and Celiac Disease: Prevalence, Diagnostic Tools and Therapy. World J. Methodol..

[67] Roshanzamir N., Zakeri Z., Rostami-Nejad M., Sadeghi A., Pourhoseingholi M.-A., Shahbakhsh Y., Asadzadeh-Aghdaei H., Elli L., Zali M.-R., Rezaei-Tavirani M. (2021). Prevalence of Celiac Disease in Patients with Atypical Presentations. Arab J. Gastroenterol..

[68] Voss J., Schneider C.V., Kleinjans M., Bruns T., Trautwein C., Strnad P. (2021). Hepatobiliary Phenotype of Individuals with Chronic Intestinal Disorders. Sci. Rep..

